# Effects of Hippotherapy on Health-Related Quality of Life in Caregivers of Children with Cerebral Palsy: A Pilot Quasi-Experimental Study in Japan

**DOI:** 10.3390/healthcare11243175

**Published:** 2023-12-15

**Authors:** Tomoko Mutoh, Tatsushi Mutoh, Hiromi Kurosaki, Yasuyuki Taki

**Affiliations:** 1 Department of Aging Research and Geriatric Medicine, Institute of Development, Aging and Cancer, Tohoku University, Sendai 980-8575, Japan; 2 Division of Clinical Psychology, Graduate School of Human Sciences, Kobe Shoin Women’s University, Kobe 657-0015, Japan; 3 Research Institute for Brain and Blood Vessels, Akita Cerebrospinal and Cardiovascular Center, Akita 010-0874, Japan

**Keywords:** animal-assisted therapy, caregivers, cerebral palsy, gross motor function, hippotherapy, quality of life, well-being

## Abstract

Background: Despite accumulating data regarding the beneficial effects of hippotherapy on gait and balance skills in children with cerebral palsy (CP), its effects on caregivers’ quality of life (QOL) are limited, presumably due to a lack of reliable and valid measurement tools. This study aims to evaluate the impact of hippotherapy on the health-related QOL of primary caregivers using the Japanese version of the Cerebral Palsy Quality of Life for Children (CP QOL) questionnaire. Methods: A quasi-experimental design embedded within our existing cohort was utilized. A total of 29 children with CP (range 4–12 years) and their caregivers participated in either a weekly hippotherapy or recreation (usual care) program for 1 year. In addition to gait-related measurements (Gross Motor Function Measure [GMFM]-E) of children, CP QOL-evidenced determinants of the caregivers’ health-related QOL and well-being were compared before and after the intervention. Results: In addition to improvements in children’s GMFM-E scores, hippotherapy improved CP QOL domains related to participation and physical health, children’s emotional well-being, and parents’ overall health (*p* < 0.05). Linear regression analysis showed a positive relationship between the children’s GMFM-E scores and their caregivers’ health domains in participants who received hippotherapy (*r^2^* = 0.404; *p* = 0.011). Conclusions: Hippotherapy has a beneficial effect on the physical and mental well-being and satisfaction of Japanese parents caring for children with CP.

## 1. Introduction

Cerebral palsy (CP), a group of permanent disorders that affect an individual’s movement and posture, is caused by non-progressive damage to the immature, developing brain [1]. Its global prevalence has been unstable among countries [estimated range of 1.5–4 (overall 2) per 1000 live births] [2] owing to the continuous rise in the number of infants born with low birth weight and differences in critical care in obstetric and neonatal practices. 

Although CP is recognized in the first year of life in the majority of severe cases, there is a delay in mild cases (usually diagnosed at around 5 years of age) between the acquisition of a cerebral lesion and its effect on motor abilities [3]. In Japan, the prevalence of CP persists at 2.27 per 1000 people (ages 5–9), even after the children’s decreased use of healthcare services [4]. Those CP children diagnosed by that age can be regarded as survivors since birth, and thus require lifelong movement therapy owing to chronic neurological abnormalities and developmental disorders. 

Ideally, a competent caregiver can not only conduct daily functional activities, but also have good behavioral management, proper stress management, and appropriate self-efficacy techniques [5]. However, taking holistic care of children with CP is both physically challenging and mentally straining for caregivers, as they require more attention while caring for their children [6,7]. Caregiving of children with CP for long periods of time can encounter negative psychological experiences during their lifetime, increasing the risk of burnout and vulnerability to physical condition and mental health status [8].

Health-related quality of life (QOL) is an integrative definition of well-being that considers a person’s physical, psychological, and social dimensions [9]. Equine-assisted therapy is a field of animal-assisted rehabilitation interventions that aims to achieve these components of well-being through physical or non-physical interactions with horses (e.g., benefits of whole-body exercise, reducing stress, and improving emotional and social skills) [10,11,12,13]. Among several types of equine-assisted therapies, hippotherapy utilizes a treatment strategy with the aid of the natural gait and movement of a horse itself, which differs from the concept of therapeutic horseback riding, where specific riding skills are taught. In particular, for children with CP, exercise-based therapies aimed at improving postural control have been used more frequently. Interestingly, recent data from a therapeutic riding center in Japan suggested that hippotherapy for children with CP, with a weekly program, promoted positive feelings among their parents, as well as facilitated children’s gait and balance abilities [14].

However, hippotherapy lacks evidence regarding its effects on health-related QOL [15], as reliable and valid tools that measure a caregiver’s self-perception of their health status, comfort, and well-being are limited. The Cerebral Palsy Quality of Life for Children (CP QOL-Child) questionnaire is a condition-specific questionnaire designed for children with CP to assess their well-being rather than ill-being [16]. It includes a parent-proxy version that aims to assess the QOL of the primary caregivers of children with CP aged 4–12 years, leading to international support for the translation and validation to establish cross-cultural applicability [17]. In fact, previous data suggest that, when using a parent proxy, the parents’ psychological state could also be measured successfully [18].

We hypothesized that if more appropriate methods could be employed, including participants and evaluation instruments specifically designed for CP, further positive effects of hippotherapy on caregivers’ QOL could be obtained. Therefore, we conducted a pilot study to evaluate the effectiveness of hippotherapy on the health-related QOL and well-being of Japanese-speaking parents of children with CP using the Japanese-translated version of the CP QOL-Child questionnaire for primary caregivers [19]. We also examined the relationship of the parents’ QOL domains with functional improvements in their children as identified by the dimension of gross-motor activities related to daily motor activities.

## 2. Materials and Methods

### 2.1. Study Design and Participants

This was a quasi-experimental design, where one group receives hippotherapy and the other group does not, embedded within our existing cohort, which included one of the two treatment groups: hippotherapy or outdoor recreation (usual care). All children aged 4–12 years at the time of enrollment and their caregivers, who could be recruited by contacting various health care, recreation, and rehabilitation facilities in northern Japan between 2017 and 2020, were eligible. The inclusion and exclusion criteria have been reported in our previous study, which was designed as a randomized controlled trial [14]. The groups were comparable in terms of the type and severity of cerebral palsy, as well as other factors such as age, gender, and comorbidities. None of the participants had previous experience with hippotherapy. The study was conducted in accordance with the Declaration of Helsinki and its future amendments. In addition, it was approved by the Society of Physical Therapy Science Ethics Committee (approval number: SPTS2016007). All experiments were performed in accordance with relevant guidelines and regulations. This includes when participants receive interventions as part of routine lessons in accordance with the standardized hippotherapy curriculum and recreational physical activity program, and a researcher studies the effect of the intervention. Written informed consent was obtained from the caregivers of all the participants.

### 2.2. Physical Therapy and Functional Evaluation of CP Children

The study was investigated at one facility (Holistic Betterment and Wellness Through Riding PIROUETTE, Tochigi, Japan) that established both therapeutic horse riding and daycare services for children with disabilities, including CP. Each program (hippotherapy or usual care) was employed for 30 min once a week for one year (48 consecutive weeks). The details of both protocols have been fully described in our previous publication [14]. Their caregivers were allowed to observe the lessons without participating in the protocol. Among of the Gross Motor Function Measure (GMFM) items, gait-related measurements (GMFM-E) were selected because this parameter was most significantly associated with a good response to hippotherapy for CP children in our previous cohort [14] and may contribute to a subjective indicator of their caregivers’ QOL. The GMFM-E were calculated in both groups by a blinded examiner before the intervention began and on the follow-up day during the cooling-off period (usually performed after one month of completion). 

### 2.3. QOL Evaluation of the Caregivers

Caregivers completed questionnaires during their visit to our healthcare facilities on the same day their children’s GMFM-E scores were assessed. The Japanese version of the CP-QOL questionnaire for primary caregivers [19] was administered to primary caregivers (parents or legal guardians). It contained 66 items that comprised nine categories (family and friends, communication, health, participation, access to services, special equipment, pain and bother, and some final questions regarding your child and your health) for the evaluation of the health-related QOL and well-being of both the children with CP and their caregivers. All items were averaged for each domain and transformed to a scale that ranged from 0 to 100, as described in the CP QOL-Child Manual [20].

### 2.4. Statistical Analysis

Data were expressed as a number (%) or mean ± SD (range). The Mann–Whitney *U* test was used to compare non-normally distributed data. Fisher’s exact test was employed. An analysis of variance (ANOVA) with post hoc Bonferroni–Dunn correction was used to compare continuous data that was normally distributed according to the D’Agostino–Pearson normality test. The linear relationship between the changes (△ from the baseline) in the statistically significant QOL domains of caregivers and children’s GMFM-E scores was visualized by scatterplots and the least-squares regression line for analyzing correlations within-group both before and after the program. Multivariate regression models were used to determine if data plots for each domain were related to improved GMFM-E scores in children who received hippotherapy.

To confirm how well the statistically significant domain(s) informed reality, an exploratory factor analysis was performed. A new structural model was identified via varimax rotation. For each item, the factor loading on the factor was produced, which indicated the correlation between the item and factor; thus, the closer to 100% covariance, the better the item. For evaluation of commonality, a value of >0.40 was considered satisfactory, while lower values suggested a small contribution to the model. All analyses were performed using SPSS (version 28; IBM, Armonk, NY, USA), Bell Curve for Excel (version 2021, SSRI, Tokyo, Japan), and Prism (version 9; GraphPad Software, La Jolla, CA, USA). The significance was set at *p* < 0.05.

## 3. Results

Demographic information of the caregiver and child pairs is presented in Table 1. All the children had spastic CP over the bilateral lower limbs. There were no significant differences in age, sex, type of CP, or children’s gross motor function classification system scores between the groups. All 29 parents (97% mothers) completed the CP-QOL questionnaire within 30 minutes. Age and educational status did not differ between the two groups.

Changes in the GMFM-E scores of children with CP and primary caregivers’ health-related QOL before and after the 1-year intervention are shown in Table 2. Hippotherapy significantly increased the GMFM-E scores of children compared with the usual care group or the baseline level, as reported previously [14,21]. In addition to improvements in children’s GMFM-E scores, we found positive effects of hippotherapy on 3 out of the seven caregiver-reported CP QOL domains related to participation, physical health, and emotional well-being of children, as well as the overall health of their parents (*p* < 0.05).

To confirm the hypothesis that hippotherapy could have a beneficial effect on caregivers’ QOL, a factor analysis was conducted. Along with improvements in children’s gross motor functions, three interpretable QOL factors were obtained (Table 3). Factors of caregivers’ subjective satisfaction, as represented by their physical and mental well-being and self-confidence in their children, accounted for 36% of the item variance, and objective factors, represented by family income, accounted for 25% (Table 4).

**Figure 1:**
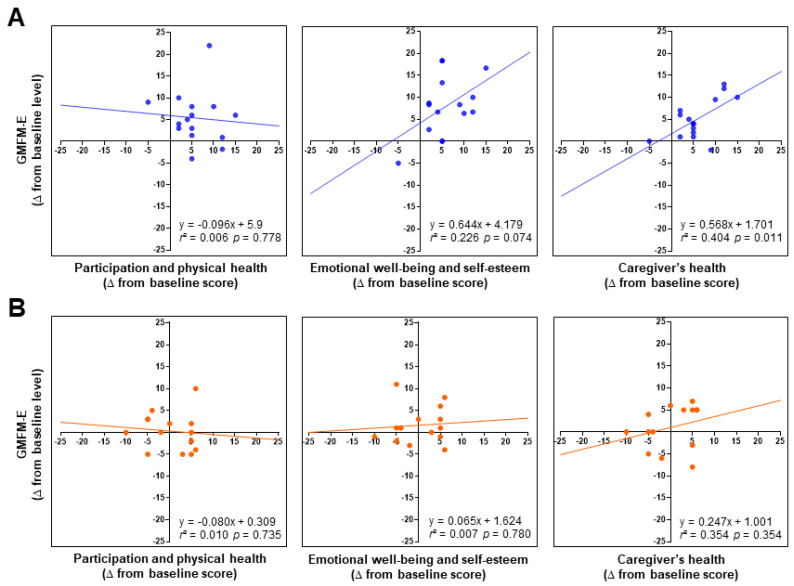
Relationships between changes (∆ from baseline) in 3 health-related QOL domain scores (**left**: Participation and physical health; middle: Emotional well-being and self-esteem; **right**: Caregivers’ health) and children’s GMFM-E levels before and after intervention with (**A**) and without (**B**) hippotherapy. Note that the scatterplots and the least-squares regression lines of the hippotherapy group show a positive statistically significant relationship between the caregivers’ health domain and GMFM-E. *r*^2^ = squared correlation coefficient.

Regarding the relationship between QOL and children’s gross motor function, linear regression analysis showed a positive relationship between GMFM-E scores and caregivers’ health domain after hippotherapy (*r*^2^ = 0.404; *p* = 0.011) (Figure 1). No other items revealed significant correlation coefficients (*p* > 0.05).

## 4. Discussion

According to the rehabilitation guidelines for patients with CP published by the Japanese Association of Rehabilitation Medicine in 2014 [22] and recent reviews [11,23], only limited studies have examined the therapeutic efficacy of hippotherapy on caregivers. Furthermore, there are no appropriate condition-specific metrics for assessing the QOL of children with CP and their caregivers simultaneously. As an alternative, we use the Japanese version of the Abbreviated World Changes in the GMFM-E scores of children with CP and primary caregivers’ health-related QOL before and after the 1-year intervention, as shown in Table 2. Hippotherapy significantly increased the GMFM-E scores of children compared with the usual care group or the baseline level, as reported previously [14,21]. In addition to improvements in children’s GMFM-E scores, we found positive effects of hippotherapy on three out of the seven caregiver-reported CP QOL domains related to participation, physical health, and emotional well-being of children, as well as the overall health of their parents (*p* < 0.05).

In this study, using the new instrument, caregivers felt satisfied with their own QOL status after completion of the 1-year weekly hippotherapy program, as well as with their children’s physical and emotional health and well-being. Interestingly, a statistically significant relationship with improved children’s GMFM-E was observed only in one of seven CP-QOL domains that reflected their parents’ self-esteem (“Your Health” domain comprised six items). Such a positive relationship of the psychological QOL domain, particularly in caregivers’ “positive feelings”, along with improvement in their children’s gait functions after hippotherapy was demonstrated in a previous study via the WHOQOL-BREF questionnaire [14]. In contrast, several reports revealed that caregivers’ health-related QOL was not necessarily related to their children’s motor function levels [24] or improvements in their level of performance [15]. Although the sample size of this study was small, which may have restricted the interpretation of the data, it was conceivable that hippotherapy had the potential to improve health-related QOL in the family caregivers of children with CP, as they recognized improvements in their children’s gait function through successful hippotherapy sessions.

Results from the factor analysis in the hippotherapy group validated our suspicion that caregivers’ satisfaction with physical and mental well-being and self-confidence in their children improved. Data also showed that family income may be another factor that influenced caregivers’ QOL. Unfortunately, the entire cost (transportation, meals, lesson expenses, etc.) of hippotherapy is not covered by social health insurance in Japan at present. Although there exists substantial heterogeneity in the population of caregivers and their family members by virtue of patterns of responses to various financial and psychological stressors [25], future efforts to help alleviate the economic burden associated with hippotherapy should also be expected.

In this study, the following limitations must be considered. First, the failure to control possible factors that may have affected the quality of life of caregivers during that year of their children’s participation in hippotherapy. Second, the fact that the sample consisted only of mothers (97%) as caregivers of children with CP. Third, this was a pilot study in Japanese, and we only had data from a single facility and a small number of participants (n = 29 pairs of CP children and their caregivers). Hence, the possibility of selection bias (e.g., family support, socioeconomic status, education level, cultural tradition, or other interventions on the QOL of children) cannot be excluded. These issues limit the credibility and generalizability of the findings and might thus not be directly applicable to other situations or to patients other than the population enrolled. Nevertheless, the application of an internationally translated CP-specific QOL measure may help extend our knowledge to understand the QOL and well-being of caregivers from a global perspective. To further explore the reliability and external validity of our results, additional work with a larger sample will be highly productive.

## 5. Conclusions

To the best of our knowledge, this is the first study to determine the beneficial effect of hippotherapy on the physical and mental well-being and satisfaction of Japanese parents caring for children with CP. 

## Figures and Tables

**Table 1:** Descriptive characteristics among children with CP and their caregivers with and without hippotherapy.

	Hippotherapy (n = 15)	Usual Care (n = 14)	*p*-Value
**Children**			
Age (years)	7.9 ± 5.8 (4–12)	8.5 ± 4.9 (5–12)	0.49
Gender (male/female)	8/7 (53/47)	8/6 (57/43)	0.57
Type of CP	Spastic, 15 (100)	Spastic, 14 (100)	
GMFCS levels			
II	6 (40)	6 (43)	0.59
III	9 (60)	8 (57)	
**Primary caregivers**			
Age (years)	36.5 ± 6.2 (28–47)	35.9 ± 5.9 (27–45)	0.79
Respondent (mother/father)	14/1 (93/7)	14/0 (100/0)	0.52
Years of education	16.3 ± 2.0 (14–18)	15.6 ± 1.8 (14–18)	0.19
Educational level			
Bachelor’s degree/Diploma	12 (80)	12 (83)	0.53
Advanced degree (Master’s or PhD)	3 (20)	2 (17)	

CP = cerebral palsy; GMFCS = gross motor function classification system categorized into 5 different levels measured by initial admission before entering the present study cohort. Data are expressed as the mean ± SD (range) or number (percentage).

**Table 2:** Health-related quality of life assessed by CP QOL-Child for primary caregivers and GMFM-E scores of CP children with and without hippotherapy.

	Hippotherapy Pre	Post	*p*-Value	Usual Care Pre	Post	*p*-Value	Intergroup *p*-Value
Social well-being and acceptance	39.4 ± 5.2	43.0 ± 7.0	**<0.001**	40.6 ± 7.3	41.9 ± 8.2	0.190	0.538
Feeling about functioning	36.9 ± 2.8	39.1 ± 5.7	0.074	38.6 ± 7.8	40.1 ± 8.5	0.193	0.560
Participation and physical health	40.5 ± 7.7	45.9 ± 7.0	**<0.001**	41.1 ± 8.0	41.7 ± 6.6	0.774	**0.038**
Emotional well-being and self-esteem	45.8 ± 3.3	53.7 ± 7.5	**<0.001**	47.1 ± 5.2	48.8 ± 5.1	0.135	**0.0013**
Pain and impact of disability	35.4 ± 6.9	34.6 ± 9.3	0.519	36.8 ± 8.9	35.7 ± 7.6	0.408	0.610
Access to services	67.5 ± 9.7	68.0 ± 08.8	0.510	68.4 ± 9.4	69.4 ± 8.6	0.255	0.578
Your health	53.3 ± 5.9	58.4 ± 5.5	**<0.001**	52.8 ± 7.4	53.9 ± 6.4	0.233	**0.010**
GMFM-E	45.4 ± 10.6	51.0 ± 9.3	**<0.001**	45.5 ± 9.0	45.6 ± 9.7	0.787	**0.044**

CP = cerebral palsy; CP QOL-Child = parent-proxy version of the cerebral palsy quality of life for children translated into Japanese; GMFM-E = walking, running, and jumping elements of the gross motor function measure. Values are expressed as the mean ± SD. Statistically significant results at *p* < 0.05 are shown in bold.

**Table 3:** Multivariate linear regression analysis of caregivers’ health-related quality of life domains for predicting improved GMFM-E scores before and after hippotherapy.

Variable	Unstandardized Coefficient		Standardized Coefficient (β)			95%Confidence Interval for *B*	
	*B*	SE		*t*	*p*-Value	Lower Bound	Upper Bound
(constant)	−1.234	2.146		−0.575	0.577	−5.958	3.491
Participation and physical health	0.215	0.174	0.258	1.235	0.242	−0.168	0.597
Emotional well-being and self-esteem	0.314	0.145	0.426	2.168	0.053	−0.005	0.633
Your health	0.685	0.246	0.612	2.781	0.018	0.143	1.227

GMFM-E, walking, running, and jumping elements of the gross motor function measure; SE = standard error. Statistically significant results at *p* < 0.05 are shown in bold.

**Table 4:** Exploratory factor analysis of a family health-related subset of the CP QOL-Child items among caregivers whose children received hippotherapy.

Factor Name	Factor	
Item	1: Subjective Well-Being (Physical and Mental Health)	2: Objective Well-Being (Income)
**Your health**		
Q62. How do feel about your physical health?	**0.661**	−0.001
Q63. How do feel about your work situation?	−0.442	0.025
Q64. How do feel about your family’s financial situation?	−0.078	**0.997**
Q65. How happy are you?	**0.561**	0.335
Q66. How confident are you that you can report how your child feels?	**0.425**	−0.080

Satisfactory values of commonality (>0.40) are shown in bold.

## Data Availability

The data that have been used are confidential to respect privacy and patient autonomy. The translated version of the CP QOL-Child parent-proxy version (for parents of children aged 4–12 years) will be provided on the request of researchers or clinicians.
